# Enhancing Learning About Epidemiological Data Analysis Using R for Graduate Students in Medical Fields With Jupyter Notebook: Classroom Action Research

**DOI:** 10.2196/47394

**Published:** 2023-05-29

**Authors:** Ponlagrit Kumwichar

**Affiliations:** 1 Department of Epidemiology Faculty of Medicine Prince of Songkla University Hat Yai Thailand

**Keywords:** learning, Jupyter, R, epidemiology, data analysis, medical education, graduate student, longitudinal data analysis, graduate education, implementation

## Abstract

**Background:**

Graduate students in medical fields must learn about epidemiology and data analysis to conduct their research. R is a software environment used to develop and run packages for statistical analysis; it can be challenging for students to learn because of compatibility with their computers and problems with package installations. Jupyter Notebook was used to run R, which enhanced the graduate students’ ability to learn epidemiological data analysis by providing an interactive and collaborative environment that allows for more efficient and effective learning.

**Objective:**

This study collected class reflections from students and their lecturer in the class “Longitudinal Data Analysis Using R,” identified problems that occurred, and illustrated how Jupyter Notebook can solve those problems.

**Methods:**

The researcher analyzed issues encountered in the previous class and devised solutions using Jupyter Notebook. These solutions were then implemented and applied to a new group of students. Reflections from the students were regularly collected and documented in an electronic form. The comments were then thematically analyzed and compared to those of the prior cohort.

**Results:**

Improvements that were identified included the ease of using Jupyter R for data analysis without needing to install packages, increased student questioning due to curiosity, and students having the ability to immediately use all code functions. After using Jupyter Notebook, the lecturer could stimulate interest more effectively and challenge students. Furthermore, they highlighted that students responded to questions. The student feedback shows that learning R with Jupyter Notebook was effective in stimulating their interest. Based on the feedback received, it can be inferred that using Jupyter Notebook to learn R is an effective approach for equipping students with an all-encompassing comprehension of longitudinal data analysis.

**Conclusions:**

The use of Jupyter Notebook can improve graduate students’ learning experience for epidemiological data analysis by providing an interactive and collaborative environment that is not affected by compatibility issues with different operating systems and computers.

## Introduction

All graduate students in medical fields must eventually learn about epidemiology. Graduate students also study essential subjects, such as research methodology and data analysis, to conduct and complete the research projects that are part of their degree requirements [1]. Studying R in an epidemiology course can help students develop important skills for data analysis, reproducibility, and collaboration, which are essential for conducting rigorous and impactful research in their field [2]. There are collections of functions that use R, known as R packages, which enhance the ability to conduct data analysis in diverse fields, such as medicine [3]. However, R packages may not be compatible with all computers or operating systems (OSs); this is often evident in the classroom environment [4].

R is a programming language–based software environment that beginners learn by studying numerous examples of command usage [5]. Teaching advanced R analysis within scheduled lecture times is not possible if compatibility issues prevent students from following along with their instructors [4]. These compatibility issues may emerge from discrepancies among various versions of R, its packages, and the OS that the student is using. These issues can lead to errors, unpredictable program behavior, or challenges in code maintenance. To minimize compatibility problems during the practicum, it is crucial that the instructor and all students use the same version of R and the packages [4]. This process must also be executed differently for Windows and Mac OSs, and there may be a diverse use of OSs among the students, including different versions of the two OSs [6]. Students may also have trouble installing packages, which requires time to fix [4]. Owing to the aforementioned difficulties, the students may be less enthusiastic about learning R [1].

Jupyter Notebook is an integrated development environment for R and Python that can function either on- or offline and allows for the blending of narrative text, mathematics, and executable code [7]. Jupyter Notebook is an open-source platform that provides an excellent learning environment for students and a better graphics interface than the original R platform [8]. Jupyter Notebook can improve the ability of graduate students in medical fields to learn epidemiological data analysis by providing an interactive and collaborative environment that allows for more efficient and effective learning [9]. By using Jupyter Notebook, students can perform interactive data analysis in R through integrated step-by-step instruction that allows them to learn data analysis easily. It also allows students to document their data analysis steps in a clear and reproducible way [10]. This can be especially important for assignments, as it allows others to follow along and understand their analysis process. Using Jupyter Notebook online can also facilitate collaboration between students and their instructors. Instructors can create and share Jupyter Notebook instances with students, and students can share their work with friends for peer review and feedback [9]. Hence, instructors can flexibly use an online Jupyter server to create interactive tutorials, assignments, and quizzes.

In our classroom, teaching R in the original version for longitudinal data analysis has often been delayed due to compatibility problems, leading to learning issues. The students were disappointed in their learning experience as computing errors and crashes during package installation prevented them from following the instructions. In this study, we collected class reflections from the students, then determined possible solutions using Jupyter Notebook. Jupyter Notebook was implemented in our classroom for the next cohort of students. This study also compared the satisfaction of the students in the original R class with the satisfaction of the students who used Jupyter Notebook.

## Methods

### Study Design

This study used action research to conduct a thematic summary of issues that were raised by the lecturer and students in the class. Action research is a form of systematic inquiry that involves educators engaging in a cyclical process of problem-solving about their practices. It is often used to improve teaching by identifying and addressing specific issues or challenges within a specific educational setting [11]. In this approach, the teacher is both the researcher and the participant, and the ultimate goal is to improve the teacher’s own practice and their students’ learning experiences. The original R version for longitudinal data analysis was used to accomplish this task. Subsequently, a detailed illustration of the solutions to the problems created through teaching the original version of R was presented using Jupyter Notebook. The solutions were implemented with a new cohort of students, and the students’ average satisfaction scores were compared with those of the previous cohort to validate the solutions’ effectiveness. This analysis identified areas for potential improvement, which can be useful in enhancing the sustainability of this approach.

### Setting and Data Source

This study was based on the longitudinal data analysis class using the tidyverse package [12]. All students had background knowledge in using Basic R and the epiDisplay package [13]. The class instruction and learning materials were shared through a circulated email system. The Department of Epidemiology, Faculty of Medicine, Prince of Songkla University (PSU) routinely collected satisfaction information from students using a web-based questionnaire (shown in Multimedia Appendix 1). The questionnaire used a five-point Likert scale and was distributed to students after class. It assessed satisfaction across five dimensions: appropriate duration, media suitability, communication skills, discussion encouragement, and critical thinking promotion. These dimensions evaluate various aspects of course satisfaction: duration pertains to time allocation for topics; media suitability measures the effectiveness of instructional materials; communication skills rate the instructor’s clarity, organization, and engagement; discussion encouragement gauges the fostering of interaction and dialogue; and critical thinking promotion examines the support for in-depth analysis and problem-solving. Higher scores in each dimension signify a more satisfactory learning experience for students.

The questionnaire was created for internal use in an arbitrary manner due to the limited number of students per annum. Consequently, no reliability study was undertaken. Routine requests were made to the students to complete the questionnaire and include their reflections on a web-based sheet after class. All data reported by the students were anonymously recorded in a secured database. This mitigated the possibility of social desirability.

### Jupyter Server Setup

In accordance with the JupyterHub guidelines [14], we established a self-hosted Jupyter server on a dedicated machine (US $8700) procured from the Division of Digital Innovation and Data Analytics (DIDA), Faculty of Medicine, at PSU. The server is equipped with a 64-core CPU and 256 GB of RAM. For the default configuration, each student was allocated a server with 1 CPU core and 500 MB of RAM. This allocation sufficed for storing their notebook and any requisite data files for the course. However, it should be noted that individual access settings can be adjusted within the server’s capacity constraints.

To initiate the server, we created a virtual machine on the DIDA server and preinstalled all necessary packages. The cost of operating this instance amounted to approximately US $20 per month, as per the university’s established rates. The management of a JupyterHub server for users necessitated that the authentication be implemented via the PSU passport service, which is provided by the Computer Center of PSU, and that resources be allocated for each user. This ensured that every student had access to essential resources without overwhelming the server’s capacity.

### Participants

With the participation of students and author PK as the teacher, the classroom action research was a collaborative learning method that changed specific actions. Participants in this study included PK and all graduate students in medical fields who were taking the longitudinal data analysis class run by PK. All students had already passed a basic epidemiology exam, so it could be inferred that they possessed a foundational understanding of epidemiological concepts and were familiar with relevant basic statistical techniques, including the R base and EpiDisplay packages. All students needed to independently analyze epidemiological data to finish their research and complete their PhD or MSc in epidemiology. The first class (class 1) was taught the original R version in October 2020, and the second class (class 2) was conducted using Jupyter Notebook in July 2022. Each class took 6 hours and comprised different students. After class finished, students from both class 1 and 2 were asked to answer the same web-based satisfaction questionnaire given by the educational assisting staff.

The intended learning outcome of both class 1 and 2 was for students to exhibit competence in using R programming for the analysis of longitudinal data. PK normally observed the action of students during each class. To facilitate individualized learning within the small class setting, students were required to independently interpret results or address parallel questions after completing exercise segments on a section-by-section basis. To further promote understanding, PK presented each student with a spontaneously devised distinct problem (improvised question) that used the same technique. For example:

- The exercise question (use “airquality” data set):
  Calculate the differences between the square root of the ozone levels on the adjacent days.
- The improvised question (use “airquality” data set):
  Calculate the differences between the cube root (change function) of the sulfur dioxide levels (change variable) for 2 consecutive days with a lag of 2 (day lag=2).

This approach ensures that students do not merely replicate the code provided in instructions but rather gain a comprehensive grasp of the material.

### Problem Identification and Solution

PK noted the problems that occurred and retrieved the comments reported by the students in class 1 from the database. The notes and comments were thematically analyzed to create the problem list. The problems were reviewed and used to develop the R Jupyter for the instruction of longitudinal data analysis. The R Jupyter content was developed incrementally to solve the problems, and subsequently, a flipped class [15] assignment was included as a preclass assignment as group work. The flipped class assignment may introduce bias due to the confusing effects of using Jupyter R Notebooks. However, it is impossible to avoid since it was mandated by the university in 2022. This enabled the students to exchange ideas through the web-based platform and collaboratively prepare for the longitudinal data analysis class.

### Implementation and Evaluation

PK created a mitigation plan for class 2, which included the development of the Jupyter Notebook (see our GitHub [16]) and PDF instruction file (see Multimedia Appendix 2). These materials were distributed to students 2 weeks prior to the commencement of the class. The students were allowed to use the Jupyter server using their PSU passport account [17]. The problems detected during class 2 were noted by PK. The anonymized satisfaction scores and comments from students were sent to PK a week later. Additional details of the average age and sex distribution of the students were attached; however, those were not linked with the scores to protect personal data.

### Analysis

This study used a thematic analysis to examine the notes and comments made by PK, which were provided by students in class 2, and compare them with thematic issues in class 1. In addition, descriptive statistics were used to compare satisfaction scores between class 1 and class 2 by ignoring parametric assumptions due to the small sample size. Opportunities for improvement were identified based on the observations made in the notes and comments gathered by PK, which were not previously observed in class 1.

### Ethics Approval

This study was approved by the Human Research Ethics Committee, PSU (REC 66-104-18-1), which authorized a waiver of consent.

## Results

### Differences Between Class 1 and 2

Table 1 summarizes the characteristics of students in two different classes. Class 1 had 9 students with a mean age of 32.9 years, while class 2 had 8 students with a mean age of 30.9 years. Both classes had a similar number of male and female students. Before starting, class 2 was given a link to access a Jupyter server and a password for internet access, and students were allowed to use any device to connect to the classroom’s wireless internet. All students chose to use their laptop.

**Table 1:** Differences between class 1 and 2.

Demographics				Class 1		Class 2
**Students’ characteristics**						
	Students, n			9		8
	Age (years), mean (SD)			32.9 (7.2)		30.9 (6.3)
	**Sex, n**					
		Male	5		5	
		Female	4		3	
**Requirement before starting the class**						
	Basic knowledge			R base and EpiDisplay		R base and EpiDisplay
	Material provided			R script file		Jupyter Notebook file and PDF file for instructions to access and use the Jupyter server
	Internet			Not required		Required
	Computational tool			Laptop computer without internet connection		Any device that can connect to the wireless internet in the classroom
	Preclass assignment			None		Flipped classroom assignment
**Intended learning outcome**						
	Outcome			Demonstrating proficiency in applying R programming for longitudinal data		Demonstrating proficiency in applying R programming for longitudinal data
	Evaluation			Active engagement in class discussions and independent problem-solving		Active engagement in class discussions and independent problem-solving; flipped classroom assignment

### Problem Identification in Class 1 and Mitigation Plan

Table 2 presents a list of thematic issues that arose during class 1, along with their corresponding mitigation plans. It also outlines particular feedback provided by students in class 1 that had to be addressed before commencing class 2.

**Table 2:** Problem identification and solution.

Thematic issue from class 1			Mitigation plan
**Author PK’s note**			
	Experiencing difficulty in installing packages	All packages would be installed in the Jupyter R server before class starts.	
	Delays in class due to unexpected errors	All codes for instruction should be tested in the Jupyter R server. All errors should be fixed before sending the material to the students.	
	Insufficient student participation	A mini-quiz will be actively assigned to students after each instruction and its example.	
**Reflections from the students**			
	Being unable to keep up with the pace of instruction due to the fast-paced environment	A Jupyter R file will be provided to the students with step-by-step instructions in a PDF file. Students could try all codes in the instruction by themselves before the class.	
	Difficulty in comprehending the analysis	A preclassroom assignment should be assigned to students as group work, so they could help each other to prepare for the class.	
	Lack of resources to support advanced materials	The GitHub link [18] of PK’s work should be provided to students after finishing class to ensure continuous learning through real-world data.	

### Comparison Between Class 1 and 2

Table 3 presents feedback on the use of Jupyter R for instruction and improvement in class 2 problems, as well as comments from the students regarding their feelings about the changes in the class.

**Figure 1:**
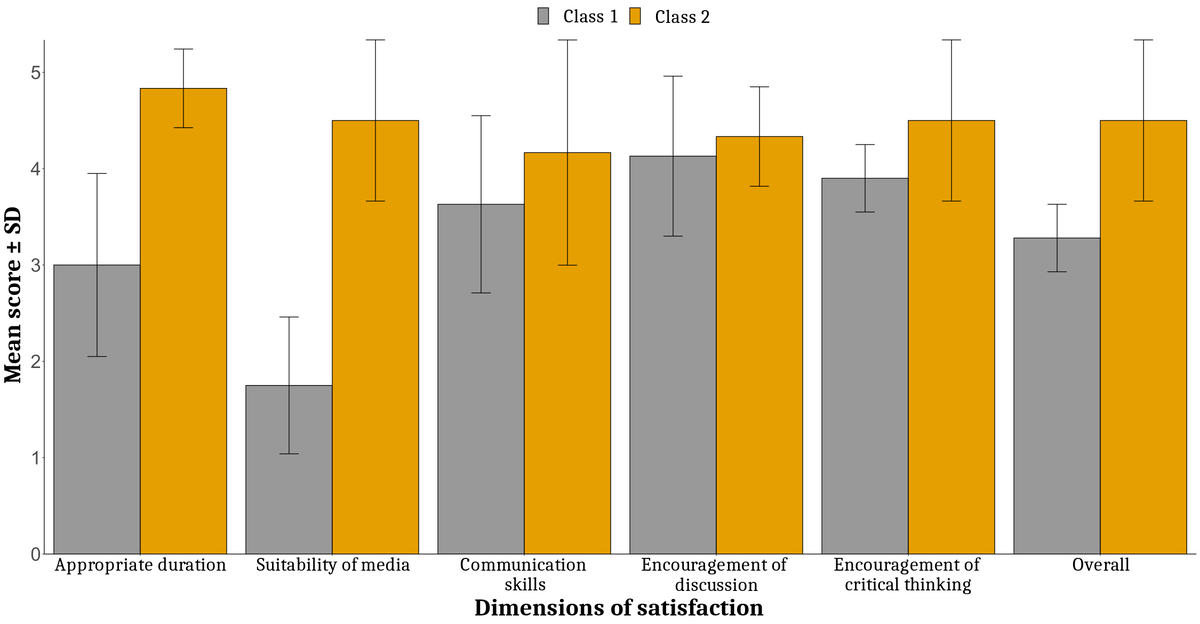
The six-dimensional satisfaction scores.

Figure 1 shows the satisfaction ratings of the two classes (class 1 and class 2) across different dimensions of satisfaction related to their learning experience. Overall, the findings suggest that class 2 (using Jupyter R) was more effective when compared to class 1, as clearly shown by the higher mean ratings and lower variability in the ratings for class 2. The explicit suitability of media in class 2 was found to be higher than that in class 1.

**Table 3:** Improvement after the use of Jupyter R for instruction.

Class 1 problems		Notes/comments supporting improvement in class 2
Experiencing difficulty in installing packages		Without needing to install packages, all students were able to immediately use the Jupyter R server for their data analysis. (author PK’s note)
Delays in class due to unexpected errors		The students were able to run all the example codes provided in the instructions without errors. However, when they attempted to code their own solutions, they encountered errors. (PK’s note)
**Infrequent student questioning**		
	PK’s note	All students asked questions frequently because they were curious about the solution for the preclass assignment and mini-quizzes.
	Comments from students	“My interest in the course was stimulated by the lecturer.” “The lecturer challenged students to do their best work and answer questions in class.” “The lecturer stimulated discussions and responded to questions.”
Being unable to follow due to the fast-paced environment		All students could try all functions of the codes through the instruction material on their own without any delay from computer-compatibility errors. (PK’s note)
Difficulty in understanding the analysis		“This subject equipped me with a comprehensive understanding of advanced statistical techniques, which will be beneficial in analyzing and interpreting the results of my research project.” “The practical exercises and case studies in this subject allowed me to apply the concepts and techniques taught in class, enhancing my data analysis skills and increasing my confidence in working with large and complex data sets.” “This subject emphasized the importance of integrating statistical analysis into research projects and providing a framework for approaching data analysis in a scientifically rigorous manner. The lessons and exercises in this subject have prepared me to effectively apply my statistical knowledge and skills to my own research project, leading to robust and reliable results.” (comments from students)
No source for the continuation of advanced practicing		“The lecturer diversified the learning level. I can learn more through Jupyter Notebook in the GitHub repository.” “I also learned to design my own research and built my analysis plan based on the example in GitHub.” (comments from students)

### Opportunities for Improvement

Table 4 presents the opportunities for improvement in class 2 and notes/comments from both the instructor and students. PK noted that there was uncertainty regarding the long-term effectiveness of the course, as there was no standard procedure in place to monitor whether students continued to engage with coding in R after class was completed. Furthermore, there is also a lack of knowledge regarding their proficiency in solving coding challenges independently. Despite PK’s emphasis that students are able to use Jupyter Notebook when they face compatibility issues related to traditional R (we also provided a Jupyter server for the alumni), it is unclear whether students would remember this or if they would use another program altogether to conduct their data analysis. Hence, a plan was developed to devise a system that could effectively monitor the adherence of students to the practice of coding in R, such as the R Skill Challenge Activity, through the Jupyter server. The student reflections highlight the need for a teaching assistant during class, as some practice sessions run slower when students require specific help. Even though the lecturer was able to cover all the necessary material with the students within the scheduled time by using Jupyter Notebook and teaching at a new pace, the students appeared to be unwilling to wait for assistance in resolving an error while the lecturer was assisting another student. Therefore, a few statisticians will be assigned as teaching assistants in upcoming classes.

**Table 4:** Opportunities for improvement.

Opportunities for improvement in class 2	Notes/comments from class 2
Uncertainty regarding the long-term effectiveness	Currently, we do not have a standard procedure in place to monitor the extent to which students continue to engage in R coding after class. Additionally, we lack knowledge regarding their proficiency in independently solving coding challenges in advance. (author PK note)
Teaching assistantship	“We have no teaching assistant during the class, and it made some practice sessions run slower because some of us encountered specific problems during the practice.” “It would be nice if teaching assistants can join these courses to help us during the practice session.” (comments from students)

## Discussion

### Principal Findings

This study focused on the challenges faced in teaching R programming in epidemiology classes and proposed the use of Jupyter Notebook as a potential solution. The study aimed to evaluate the effectiveness of Jupyter Notebook in a longitudinal data analysis class and collected reflections from students in a previous class regarding the problems they encountered in learning R programming. The findings of the study indicated that Jupyter Notebook could provide an interactive and collaborative environment that improves the effectiveness and efficiency of the learning process.

Reflections on the action research process revealed that compatibility issues and package installation crashes were the most common challenges faced when teaching R programming. These challenges were resolved by using Jupyter R Notebook, which also facilitated group work and collaborative learning. This study is innovative in its use of Jupyter Notebook as a pedagogical tool for the instruction of epidemiology and, to the best of the author’s knowledge, is the first study to do so. However, previous studies in other fields [9,19-22] have revealed that Jupyter Notebook is an effective tool for teaching data analysis.

The primary strength of this study was its collaboration with students, allowing their problems to be identified so that solutions could be found to address those issues. Moreover, the use of Jupyter Notebook as a tool to enhance learning is an innovative approach to teaching epidemiology. The use of this tool was a pragmatic remedy to the obstacles encountered when instructing students in R programming within epidemiology courses. Jupyter Notebook provided an effective and efficient learning environment, enabling students to explore data and document their analysis steps in a clear and reproducible way. Moreover, Jupyter Notebook facilitates collaboration between students and instructors, allowing instructors to create interactive tutorials, assignments, and quizzes.

### Limitations

Unfortunately, this study’s focus on a particular class and context constrains its generalizability. Additionally, the long-term efficacy of the Jupyter Notebook method in enhancing student learning outcomes remains unreported. Future research should assess the long-term effectiveness of the Jupyter Notebook strategy in augmenting student learning outcomes. Moreover, to adhere to ethical standards during student data collection, it is crucial to establish a research protocol that delineates the process for securing informed consent prior to further evaluation. The use of a flipped classroom assignment in class 2 may have influenced the overall feedback, complicating whether the observed outcomes could be exclusively attributed to the Jupyter Notebook approach.

Considering these constraints, we propose that subsequent research should examine the long-term effectiveness of the Jupyter Notebook approach in fostering student learning outcomes while accounting for confounding factors, such as flipped classroom assignments. This will facilitate a clearer understanding of the primary effect and aid in discerning the distinct contributions of the Jupyter R notebook method to student learning.

### Conclusion

Jupyter Notebook can enhance the learning of epidemiological data analysis for graduate students by providing an interactive and collaborative environment that allows for more efficient and effective learning. The findings of this study demonstrate that Jupyter Notebook can help address the challenges of teaching R programming in epidemiology classes, which are caused by compatibility issues with different OSs and computers.

## Appendix

Multimedia Appendix 1 The web-based questionnaire for student satisfaction survey.

Multimedia Appendix 2 Jupyter Notebook instruction.

## Abbreviations

DIDA: Digital Innovation and Data Analytics
OS: operating system
PSU: Prince of Songkla University

## Data Availability

The pedagogical resources pertinent to this research are publicly accessible via the GitHub repository [16]. The data sets substantiating the outcomes of this investigation can be available from the corresponding author, contingent upon a reasonable request.
