# Supplementary material for: Enhancing Learning About Epidemiological Data Analysis Using R for Graduate Students in Medical Fields With Jupyter Notebook: Classroom Action Research
Source: JMIR Med Educ. 2023 May 29;9:e47394. doi: 10.2196/47394 (PMC10262020; doi:10.2196/47394)
Supplement: Multimedia Appendix 1 [file mededu_v9i1e47394_app1.pdf]

Supplementary Appendix 1

The online questionnaire for student satisfaction survey

Class Evaluation for class 1&2

Overview | Edit questions | Templates | Analysis | Show responses

Add question [Choose...]

1. Appropriate duration
Edit

- 1/ Needs improvement
- 2/ Fair
- 3/ Average
- 4/ Good
- 5/ Excellent

2. Suitability of media
Edit

- 1/ Needs improvement
- 2/ Fair
- 3/ Average
- 4/ Good
- 5/ Excellent

3. Communication skills
Edit

- 1/ Needs improvement
- 2/ Fair
- 3/ Average
- 4/ Good
- 5/ Excellent

4. Encourages discussion
Edit

- 1/ Needs improvement
- 2/ Fair
- 3/ Average
- 4/ Good
- 5/ Excellent

5. Encourages critical thinking
Edit

- 1/ Needs improvement
- 2/ Fair
- 3/ Average
- 4/ Good
- 5/ Excellent

Comments Edit
