# Supplementary material for: Enhancing Learning About Epidemiological Data Analysis Using R for Graduate Students in Medical Fields With Jupyter Notebook: Classroom Action Research
Source: JMIR Med Educ. 2023 May 29;9:e47394. doi: 10.2196/47394 (PMC10262020; doi:10.2196/47394)

Supplementary Appendix 2

Jupyter notebook instruction

1. go to URL: https://www.dida.psu.ac.th/

2. Click log in.

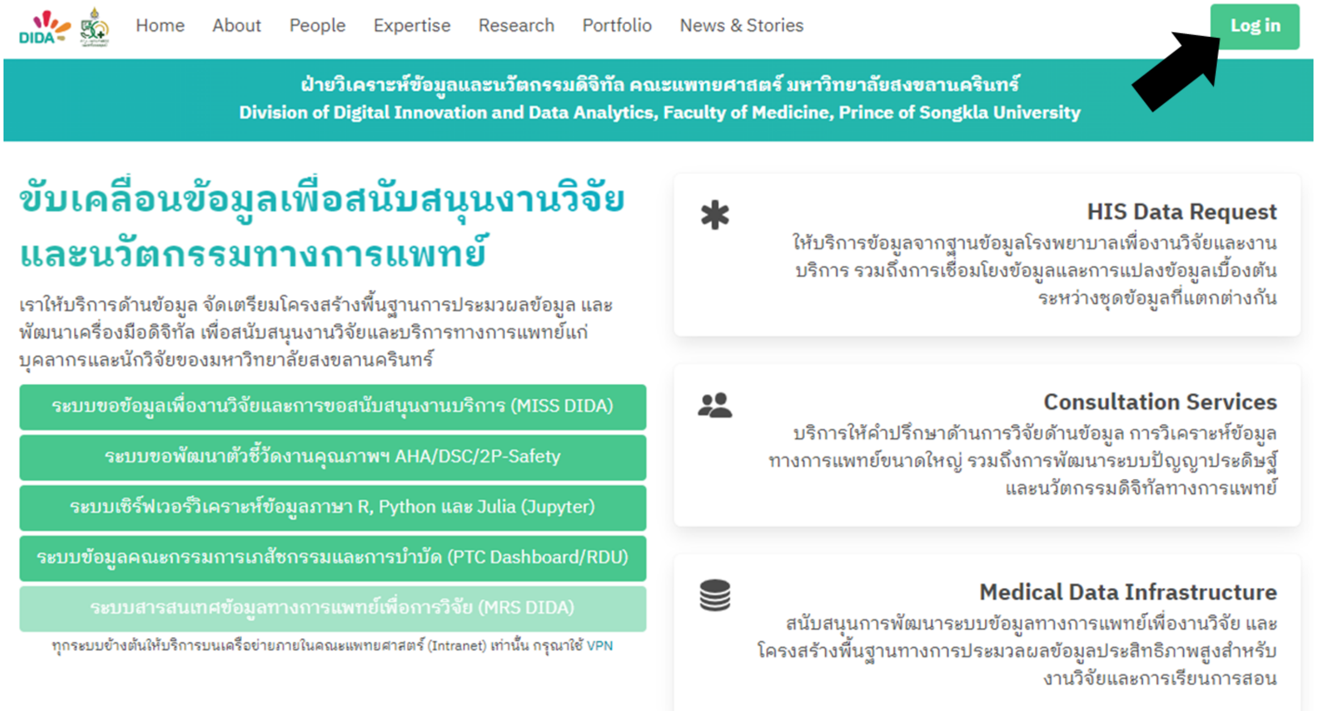

3. Log in using your PSU passport account

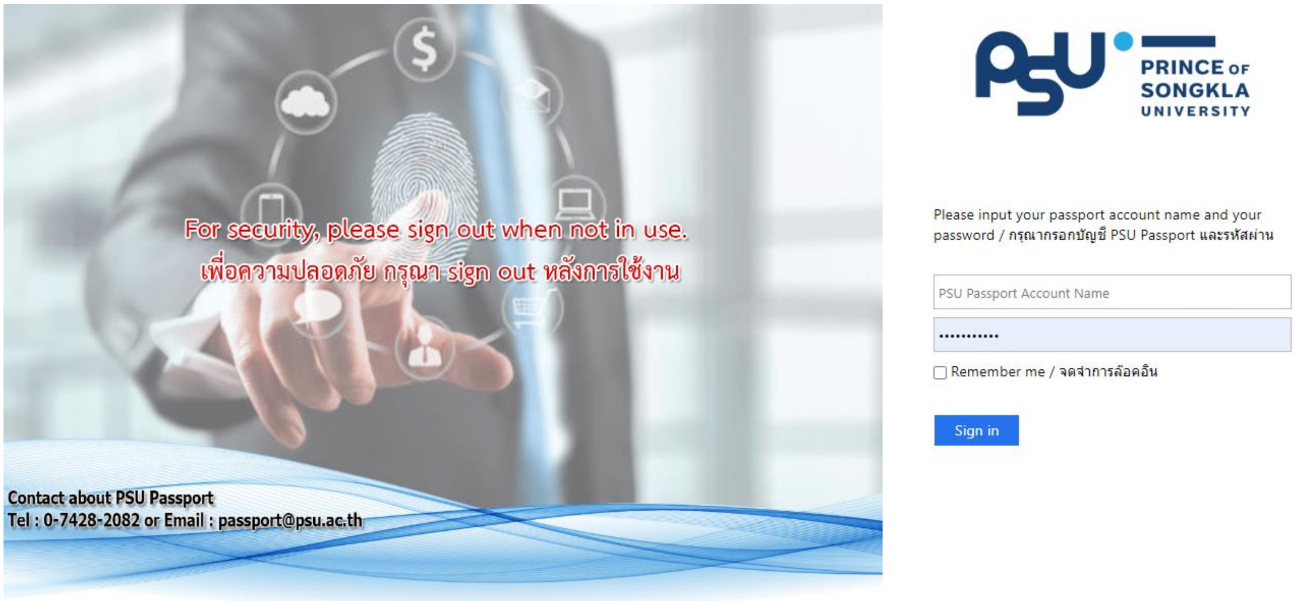

4. Read the instruction and click ‘authorize application’.

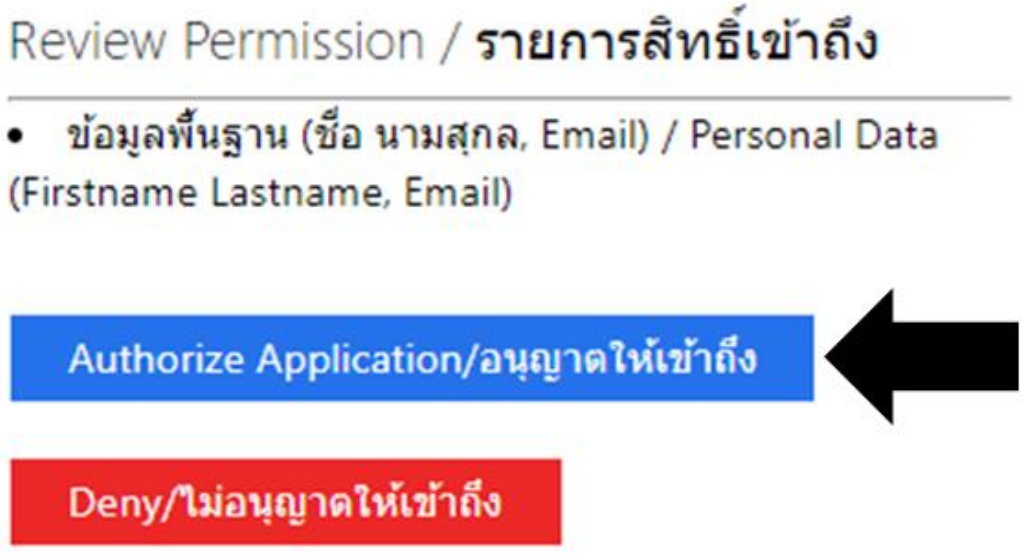

5. Go back to the home page and scroll down, then click the Jupyter server button.

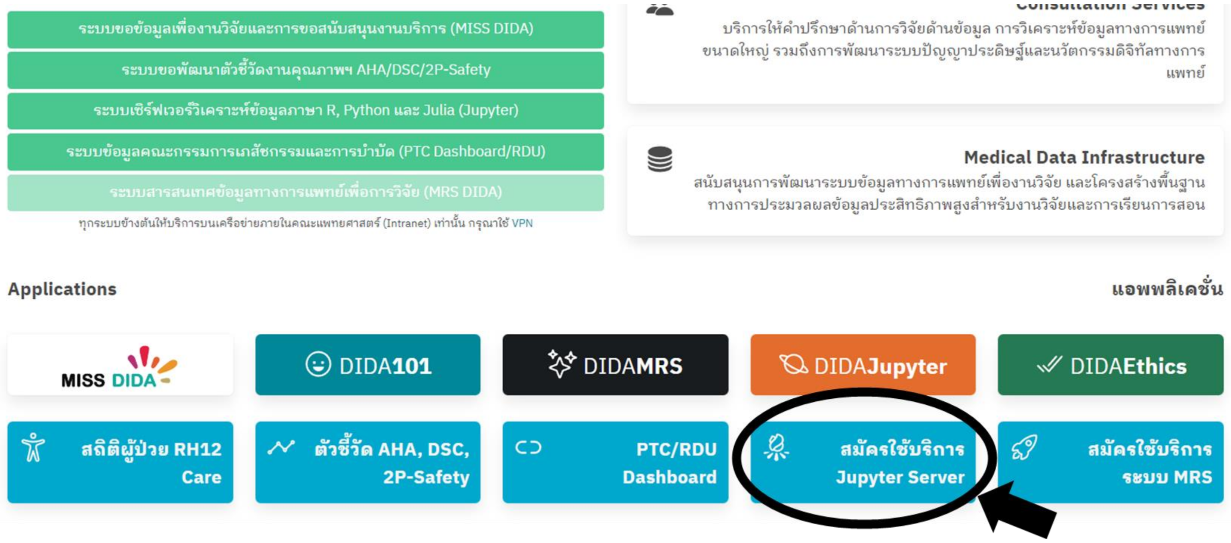

6. Read the instruction and click sign up.

Providing R, Python and Julia analytics server system on Jupyter Hub

Data Analytics and Digital Innovation Department Faculty of Medicine Prince of Songkla University provides Jupyter Server service to process data with R, Python and Julia languages for staff and students of the Faculty of Medicine. Prince of Songkhla University Users are allocated a personal container and storage space. All data will be stored in the Faculty of Medicine's server. Prince of Songkla University

What is Jupyter Notebook?
Jupyter Notebook is a programming tool for data science. Users can create and share documents containing code, equations, diagrams. Data cleanup and conversion Numerical simulation Statistical Modeling machine learning visualization Machine Learning and More

What is JupyterHub?
JupyterHub is a tool for providing Jupyter Notebooks to many users. Provide users access to the computing environment and resources without burdening users with installation and maintenance. Users include students, researchers, physicians, pharmacists and data scientists. Able to complete tasks in their own workspace using shared resources Which administrators can manage efficiently

Which is the default JupyterHub Docker Image to use?
The default JupyterHub Docker Image we use is `jupyter/datascience-notebook` . You can request another Docker Image by submitting a Support Ticket at https://miss.dida.psu.ac.th .

Who can access it?
We enable automation for staff, researchers and medical students. Prince of Songkhla University However, the terms of use must be accepted by signing the form below. Other users with PSU Passport can request access to the system as well.

Can I use RStudio?
Users can run R through their Jupyter Notebook from any computer with Internet access. What are

the Packages and Libraries installed in JupyterHub?
By default, Jupyter Notebook comes with Python 3.8, R 4.0, and Julia 1.7, along with the basic data science-related packages already installed. You can find more information about Packages and Libraries at `jupyter/datascience-notebook` , `jupyter/scipy-notebook` and `jupyter/r-notebook` Can I

install additional Packages and Libraries?
You can install additional Packages and Libraries using the standard method. without affecting other users. For Python, you can use the `!pip install package-name` or command `!conda install package-name` . For R, you can `install.packages()` use

Sign up for the Jupyter Hub service.

7. Read the instruction; tick "I understand the usage context and limitations of the system and will strictly comply with the terms of service," and click "register."

restrictions

- If you do not log in to the system for more than 7 days (168 hours), your container may be stopped. and may cause the backlog of work to be affected However, your data will not be lost. If you wish to process continuously, please login every 7 days.
- The Jupyter Server service server does not have any backup process. We are not responsible in the event that your data is lost which may be caused by force majeure such as hardware failure, power outage, or reallocation of resources, etc.

Users can access it at **https://jupyter.dida.psu.ac.th** You will be granted the license after you agree to the terms of service by signing below. For additional support requests such as adding resources, deleting containers, resetting containers, or asking for more information, please submit a Support Ticket at **https://miss.dida.psu.ac.th .**

Please ensure that the processing of your medical data is in accordance with the Personal Data Protection Act B.E. 2562 that you have signed with the Panel.

Register for the service

Username: [redacted]
Name (Thai): [redacted]
Name (English): [redacted]
E-mail: [redacted]

☑ I understand the usage context and limitations of the system and will strictly comply with the terms of service.

register

8. Go to URL: https://jupyter.dida.psu.ac.th and log in with your PSU passport account.

9. After logging in, wait for the server to load, and then click "🡅" to upload the file you received in your e-mail.

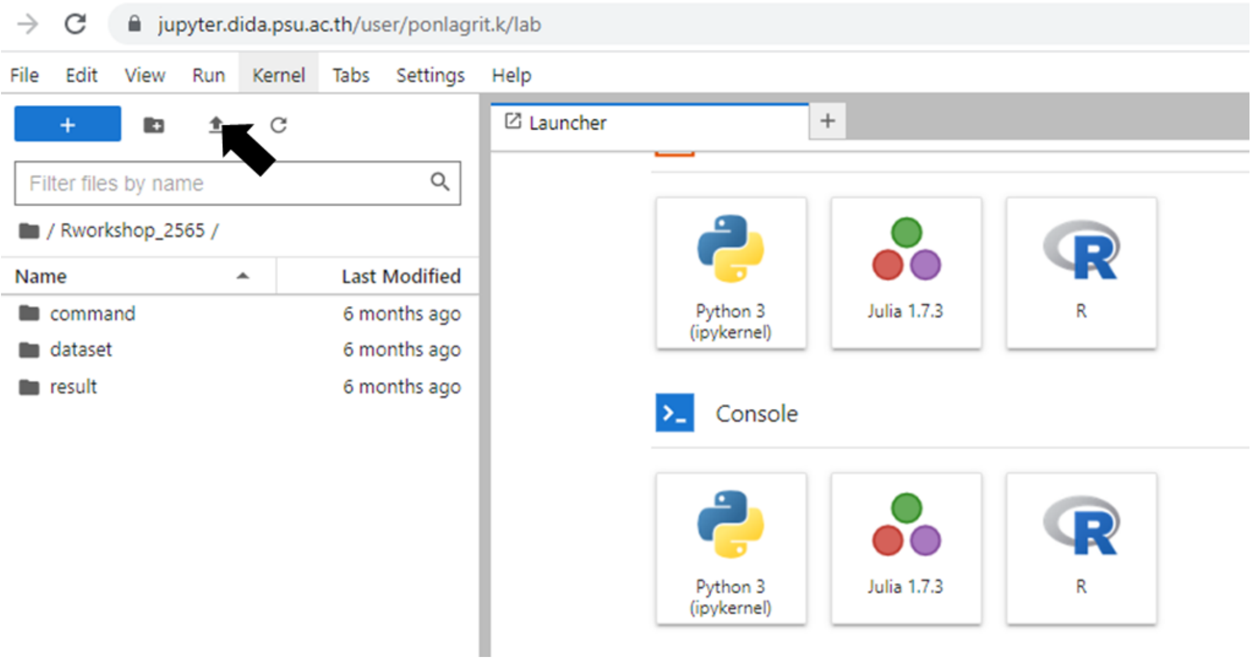

10. **Double-click** on the file uploaded.

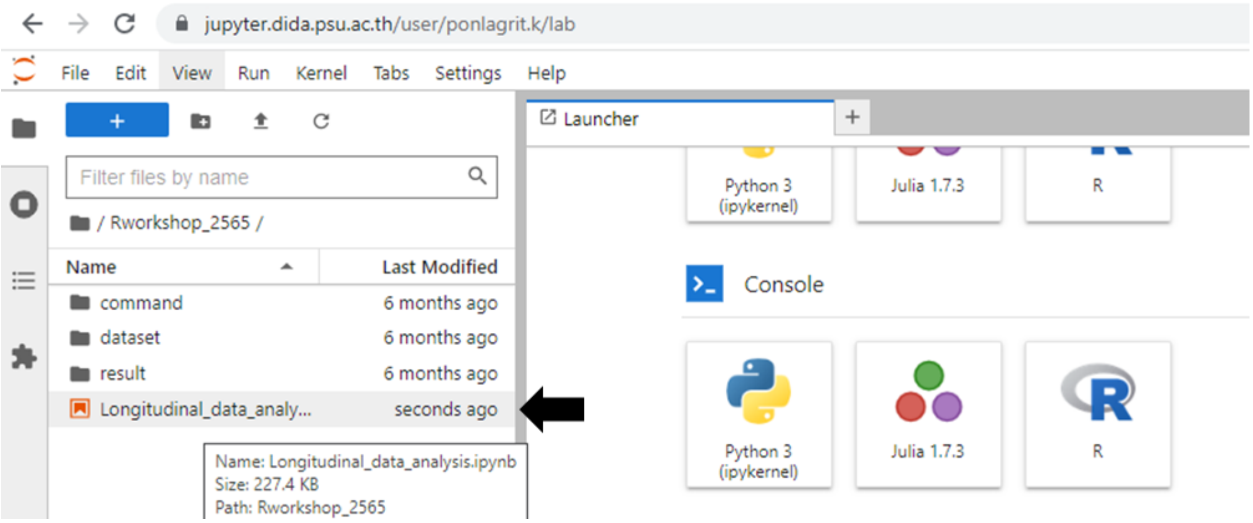

11. Click the command line and press “Ctrl” + “Enter” to run the code.
[Mac: “Command” + “Enter”s]

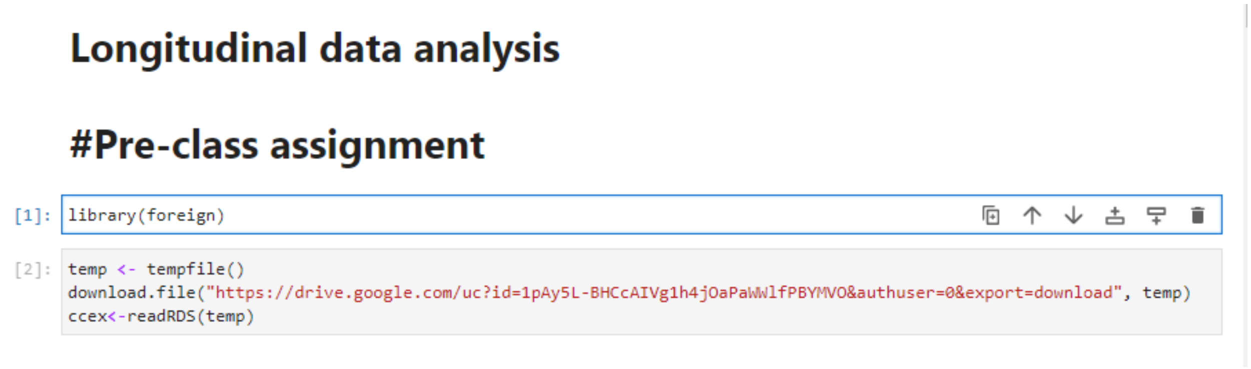

12. If you need to insert a command line, click “+”

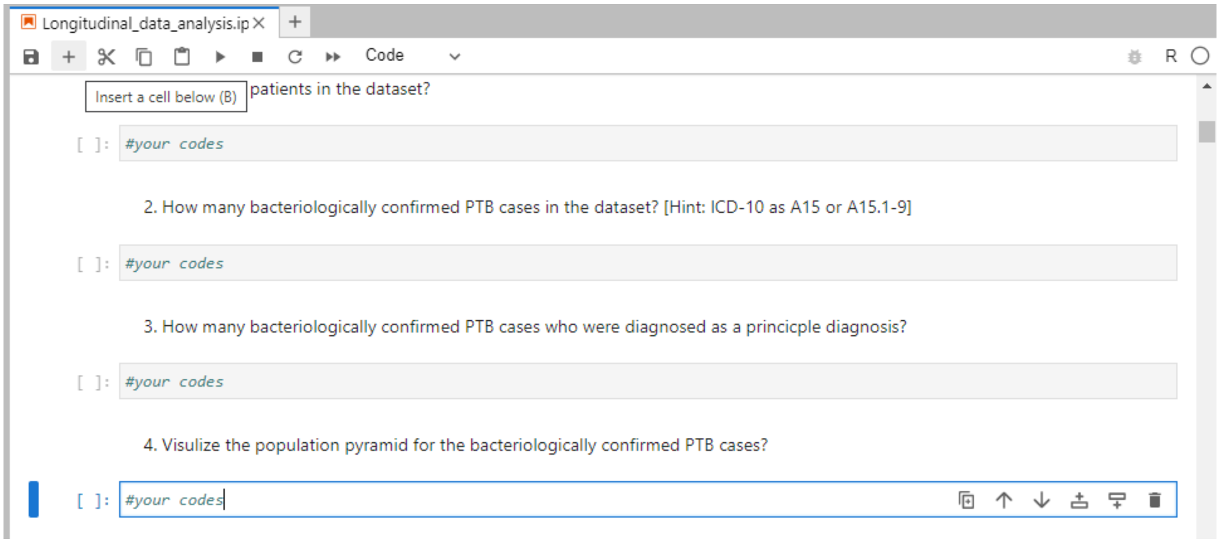

Supplement: Multimedia Appendix 2 [file mededu_v9i1e47394_app2.pdf]
